# Sodium gradient, xerostomia, thirst and inter-dialytic excessive weight gain: a possible relationship with hyposalivation in patients on maintenance hemodialysis

**DOI:** 10.1007/s11255-013-0576-y

**Published:** 2013-10-06

**Authors:** Agnieszka Bruzda-Zwiech, Joanna Szczepańska, Rafał Zwiech

**Affiliations:** 1 Department of Pediatric Dentistry, Medical University of Lodz, Pomorska 251, 92-213 Lodz, Poland; 2 Department of Kidney Transplantation, Dialysis Department, Norbert Barlicki Memorial Teaching Hospital No. 1, Medical University of Lodz, Kopcińskiego 22, 90-153 Lodz, Poland

**Keywords:** Hyposalivation, Inter-dialytic weight gain, Sodium gradient, Thirst score, Xerostomia inventory

## Abstract

**Purpose:**

The aim of the study was to assess whether hyposalivation is linked with increased thirst sensation and weight gain in hemodialysis (HD) patients and whether there is any connection between hyposalivation and sodium balance.

**Methods:**

One hundred and eleven participants (64 males and 47 females) receiving maintenance hemodialysis, mean age 59.1 ± 13.6 years old, were involved in the study. All participants completed a survey evaluating thirst intensity (DTI) and xerostomia inventory (XI). In addition, pre-dialysis sodium concentration and inter-dialytic weight gain (IWG) were assessed. The division into no-hyposalivation and hyposalivation groups was based on an unstimulated whole saliva (UWS) flow rate.

**Results:**

Hyposalivation, UWS below 0.1 mL/min, was reported in 28.8 % of HD patients. In these participants, IWG was higher than in patients with UWS > 0.1 mL/min (3.65 ± 1.78 vs 3.0 ± 1.4; *p* = 0.042), as well as the pre-dialysis sodium gradient (3.22 ± 2.1 vs 1.6 ± 2.8; *p* = 0.031). The mean XI and DTI scores did not differ between study groups. In the hyposalivation group, pre-dialysis sodium serum gradient negatively correlated with saliva outflow (*ρ* = −0.61, *p* = 0.019) and positively with IWG (*ρ* = 0.49, *p* = 0.022). IWG correlated with XI (*ρ* = 0.622, *p* = 0.016) in hyposalivation group and with DTI in no-hyposalivation group (*ρ* = 0.386, *p* = 0.033).

**Conclusions:**

Hyposalivation significantly correlates with IWG; however, its influence on thirst and self-reported mouth dryness seems to be weaker than expected. Additionally, hyposalivation was found to be associated with an elevated pre-dialysis sodium gradient.

## Introduction

Patients with end-stage renal disease treated with intermittent hemodialysis (HD) have to maintain proper fluid volume balance, which should be achieved by daily restrictions in fluid consumption [1]. The improper drinking behaviors seen in this group of patients leads to chronic fluid overload, which may result in uncontrolled hypertension, pulmonary edema or other cardiovascular manifestations, and dramatically increase the risk of premature death [2]. Thus, although inter-dialytic weight gain (IWG) seems to be an indirect indicator of patients’ adherence to the renal replacement therapy, it may be modulated by many factors [3], the foremost being excessive thirst, probably stimulated by xerostomia (a feeling of a dry mouth) [4]. In addition, some hemodialysis patients may demonstrate impaired saliva secretion, which not only produces an oral cavity environment conducive to caries associated with changes in oral soft tissue (e.g., mucosal soreness, gingivitis, cheilitis fissuring of the tongue and recurrent yeast infections) but may also enhance thirst and a subjective sensation of a dry mouth [5–10]. Bots et al. [4] note that these factors contribute to the intake of fluids and consequently to excessive IWG in patients on maintenance hemodialysis.

Additionally, our previous study demonstrates that thirst and IWG may not be linked with pre- nor post-dialysis sodium serum concentration, but mainly with pre-dialysis sodium gradient [11], which makes this factor worthy of further consideration.

The aim of the study was therefore to determine whether hyposalivation is really a factor which enhances xerostomia, thirst and weight gain (IWG) in patients on maintenance hemodialysis. The study also tries to establish a connection between hyposalivation and sodium balance.

## Material and method

A prospective trial was conducted in 111 maintenance hemodialysis patients (64 males and 47 females), mean age 59.1 ± 13.6 years. The mean time from starting hemodialysis was at least 6 months: The mean time being 14.7 ± 8.9 months. All subjects were recruited from the Dialysis Department of the Norbert Barlicki Memorial Teaching Hospital No. 1. The mean session time was 253 min. The causes of end-stage renal disease included chronic glomerulonephritis in 28 patients, diabetic nephropathy in 40, adult polycystic kidney disease in 8, hypertension in 16, tubulointerstitial nephritis in 6 and unknown in 13 patients. The eligibility criteria for a patient to be included into the study were as follows: age between 18 and 80 years old, a fixed hemodialysis schedule of 3 times a week and a stable clinical condition. The exclusion criteria comprised uncontrolled hypertension or recurrent symptomatic hypotension episodes, chronic heart failure (NYHA stage 4), severe acute infections requiring hospitalization and the administration of centrally acting sympatholytics. All patients were advised to maintain their usual dietary habits.

Of the participants, two subgroups were formed basing on the presence of hyposalivation, defined by a salivary flow rate below 0.1 mL/min [12]. To confirm or exclude hyposalivation, unstimulated whole saliva (UWS) was collected for 5 mins through use of the spitting method before a mid-week HD session. The subject refrained from eating, tooth brushing, mouth rinsing or smoking for at least 1 h before spitting. They were seated in upright position and asked to relax during spitting. The participants were instructed to avoid swallowing the saliva during sample collection to allow the saliva to accumulate in the floor of the mouth and were instructed to spit out into test tubes every 30 s for 5 mins. The saliva flow rate was then calculated to milliliters per minute.

All participants also completed a survey evaluating thirst intensity and xerostomia. The dialysis thirst inventory (DTI) is a questionnaire which consists of 7 items, while the validated xerostomia inventory comprises 11 items, each with a 5-point Likert scale ranging from never (1) to always (5). The results of the inventories range from a minimum of 7 and 11 points (no thirst and no dry mouth) to a maximum of 35 and 55 points (enormous thirst and extremely dry mouth), respectively. Both questionnaires were conducted together with the biochemical tests, i.e., pre- and post-dialysis serum sodium concentration and sodium gradient: The difference between serum sodium and dialysis fluid sodium concentration presented as absolute numbers. All measurements were carried out routinely in certified central hospital laboratory automatic analyzers. Simultaneously, IWG, defined as the difference between current body mass and dry weight (IWG), and blood pressure (BP) were measured. All assessments, i.e., blood specimens and saliva collection as well as the survey, were conducted with the principle of the single time point assessment (a mid-week HD session).

The antihypertensive treatment allowed BP below 140/90 mmHg before and 130/80 mmHg after hemodialysis to be achieved in most of the participants. In both subgroups, antihypertensive treatment was not changed and doses were stable.

The kidney replacement therapy was conducted on Fresenius 4008 dialysis machines exclusively. Standard bicarbonate dialysate fluid containing 140 mmol/L of sodium, 1.25 mmol/L of calcium and 0.75 mmol/L of magnesium was used. The potassium concentration varied depending on the degree of the patient’s kalemia before the session. The dialysis adequacy was assessed with a single pooled *kT*/*V* of average value 1.2–1.4. The dry weight was established based on clinical examination, BP measurements and whole body composition spectroscopy [13].

In all participants, the mineral bone disorder associated with their renal anemia and kidney diseases was successfully treated according to KDOQI recommendations [14, 15] as was diabetes mellitus [16]. Both study subgroups were age and sex matched, and significant parameters were comparable with regard to the number of participants. The characteristics of subgroups are summarized in Table 1.

**Table 1:** Characteristics of the study group

	Hyposalivation	No-hyposalivation	*p* value
*N*	32	79	NS
Males	19	45	NS
Age (years)	59.1 ± 14.2	58.3 ± 13.5	NS
Diabetes (*n*)	15	31	NS
HbA1c (%)	6.3 ± 0.4	6.4 ± 0.3	NS
Smokers (*n*)	8	11	NS
Hemodialysis vintage (months)	13.8 ± 7.2	14.2 ± 6.9	NS
Dialysis session time (min)	255 ± 20	250 ± 30	NS
*kt*/*V*	1.21 ± 0.2	1.22 ± 0.15	NS
Hgb (g/dl)	10.8 ± 1.5	10.7 ± 1.3	NS
Albumins (g/L)	4.0 ± 1.9	3.9 ± 2.1	NS
Residual diuresis (*n*)	9	17	NS
Volume (mL/day)	740 ± 120	710 ± 110	NS
ACEi treatment (*n*)	18	35	NS
Xerogenic medication (*n*)	7	13	NS
Alcohol consumption	1	3	NS
Dentures (*n*)	12	26	NS

*HbA1c* glycosylated hemoglobin type A1c, *Hgb* hemoglobin

Values are mean ± standard deviation (SD)

*NS* not significant

### Statistical analysis

The abnormality of distribution was checked by the Kolmogrov–Smirnov test. Comparisons between the study subgroups were performed using the Mann–Whitney test. The Fisher’s exact probability test was used for gender comparison. Correlations were assessed by Spearman’s rank correlation coefficient. Associations between IWG and pre-, post-dialysis sodium gradient or serum concentration, xerostomia, thirst score, and hyposalivation were estimated by using generalized linear regression with a compound symmetry covariance structure.

Differences were considered significant if *p* was less than 0.05. The results were expressed as mean ± standard deviation. Statistical analysis was performed using Statistica for Windows software (version 10.0).

The study was conducted in compliance with the principles of the Helsinki Declaration. The study protocol was approved by the Medical University of Lodz Bioethics Committee, Resolution Number RNN 147/09/KE. According to principles of GCP, informed consent was obtained from all patients prior to their inclusion in the study.

## Results

### Saliva flow rate and IWG

The mean unstimulated salivary flow was 0.31 ± 0.28 mL/min. Hyposalivation (UWS < 0.1 mL/min) was reported in 28.8 % of HD patients. A statistically significant difference was seen between subgroups with regard to inter-dialysis weight gain, which was higher in participants with hyposalivation (Table 2).

**Table 2:** The comparison of parameters in patients with and without hyposalivation

	Hyposalivation	No-hyposalivation
Pre-dialysis sodium serum concentration (mmol/L)	136.9 ± 2.4	138.3 ± 2.8
Post-dialysis sodium serum concentration (mmol/L)	138 ± 2.6	138.4 ± 2.2
Pre-dialysis sodium gradient	3.22 ± 2.1*	1.6 ± 2.8*
Post-dialysis sodium gradient	1.9 ± 2.4	1.5 ± 2.2
Thirst score (pts)	17.9 ± 5.9	18.5 ± 6.9
Xerostomia score (pts)	34.1 ± 11.0	31.7 ± 11.3
Inter-dialysis weight gain (kg)	3.65 ± 1.78**	3.0 ± 1.4**

Values are mean ± standard deviation (SD)

* *Z* = 2. 9, *p* = 0.0314

** *Z* = 2.73, *p* = 0.0424

### Sodium serum concentration and its gradient

Both patients with and without hyposalivation demonstrated similar post-dialysis sodium serum concentrations. Although the pre-dialysis sodium serum concentration was lower in the subgroup with hyposalivation than the one without, the differences did not reach statistical significance (Table 2). Similarly, although the post-dialysis sodium gradient in both subgroups did not differ, the pre-dialysis gradient was significantly higher in the hyposalivation subgroup (Table 2).

**Fig. 1:**
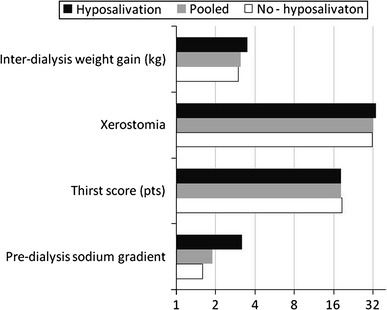
The comparison of assessed parameters in subgroups and in pooled HD patients presented as a graph with logarithmic scale

The pre-dialysis sodium gradient in both subgroups in comparison with pooled HD patients is presented in Fig. 1.

In the hyposalivation group, pre-dialysis sodium serum gradient negatively correlated with saliva outflow (*ρ* = −0.61, *p* = 0.019) and positively with IWG (*ρ* = 0.49, *p* = 0.022). In no-hyposalivation subgroup, no correlations were noted.

### Thirst and xerostomia scores

The mean xerostomia score of the study population was 33.1 ± 10.7. No statistically significant differences were found between the subjects with hyposalivation and the rest of HD patients (Table 2). The mean DTI score was 18.6 ± 6.21. Interestingly, as with the xerostomia inventory, the thirst scores showed little variation between the hyposalivation and no-hyposalivation subgroups (Table 2).

The results in both subgroups and in pooled HD patients are presented on Fig. 1.

### Correlations between IWG, xerostomia, thirst and saliva flow rate

A positive correlation between IWG and xerostomia (*ρ* = 0.341, *p* = 0.038), as well as a low and positive correlation between IWG and thirst (*ρ* = 0.2, *p* = 0.041), were observed in all HD patients.

Although positive correlations between thirst score and IWG was noted (*ρ* = 0.386, *p* = 0.033) in the no-hyposalivation group, no significant correlations were found in the hyposalivation group, except between IWG and xerostomia score (*ρ* = 0.622, *p* = 0.016). No correlations between unstimulated salivary flow rate and IWG, thirst inventory or xerostomia score were found, neither in the whole group of patients nor in the subgroups of patients with hyposalivation.

### Multivariable analysis

In multivariable analysis, pre-dialysis sodium and saliva flow rate remained significant predictors of IWG. No interactions were present between other variables and IWG (Table 3).

**Table 3:** Multivariable predictors of excessive weight gain in hemodialysis patients (whole cohort)

	Estimation	Odds ratio	95 % CI	*p* value
Pre-dialysis sodium serum concentration	−0.06	0.74	0.81–1.11	NS
Post-dialysis sodium serum concentration	−0.21	0.98	0.59–2.5	NS
Pre-dialysis sodium gradient	0.96	0.39	0.38–1.5	<0.01
Post-dialysis sodium gradient	0.06	0.51	0.24–1.11	NS
Thirst score	0.21	0.88	0.12–2.6	NS
Xerostomia score	0.29	1.9	0.85–3.31	NS
Saliva flow rate	−1.98	2.2	0.7–4.31	<0.01

## Discussion

According to the most recent criteria, impaired saliva secretion, hyposalivation, is defined as unstimulated salivary flow rates below 0.1 mL per/min [12, 17]. The percentage of patients treated with intermittent hemodialysis, in whom objectively measured hyposalivation was observed to be 28.8 %, which was lower than that found by Bots et al. [4] who note decreased salivation in 36.2 % of cases. However, different criteria were used by these authors to define hyposalivation, a 0.15 UWS flow rate, which would have widened the group of patients, and the subjects of the present study were treated in one center and lived in one region, in contrast to the multi-center study performed by Bots et al. [4]. The mean HD vintage time in our study was relatively shorter than in other studies; however, the study group was more homogenous in regard to this parameter range (6–54 months) than in cited reference (range 3–188 months) [4]. It might be possible that the relatively short hemodialysis vintage is reflected lower than in Bots et al. study percentage of HD patients with hyposalivation. But, on the other hand, according to Bots et al. [18], after 2 years of follow-up, no change from baseline for UWS value was noted in patients who remained on dialysis (0.31 ± 0.19 vs 0.31 ± 0.18 mL/min). Additionally, in the study by Kho et al. [6], the HD vintage was shorter than in both of Bots et al. studies (22 vs 35.8 and 33 months) [5, 18], but the mean UWS values in those trials were comparable.

The percentage of HD patients with hyposalivation is higher than in general population. Wiener et al. [17] determined the percentage of older adults with diagnosed hyposalivation (UWS < 0.1 mL/min) to be 12.1 %, which is over two times lower than in participants of our study, even though the population of older adults (over 70 years old) is susceptible to reduced saliva production related to certain medications and chronic conditions.

However, although the mean salivary flow in our study (0.31 ± 0.28 mL/min) was slightly higher, it was still comparable with the mean salivary flow rates demonstrated in Bots et al. (0.30 ± 0.22 mL/min) or Galvada et al. (0.28 ± 0.16 mL/min) [4, 5]. The unstimulated salivary flow rate in the present study was close to the value obtained by Wiener et al. [17] for a population of older adults (0.4 ± 0.3 mL/min). Our study also seems to confirm the finding that unstimulated salivary flow rate is comparable with values for healthy subjects [4, 5]. However, different results were obtained by Kho et al. Despite the fact that the average UWS flow rate was very similar to the values given in the studies mentioned above (0.30 ± 0.18 mL/min), those authors found it to be significantly different to the UWS flow rate of their control group (0.45 ± 0.25 mL/min) [6].

Xerostomia, defined as the subjective sensation of oral dryness, is an important condition that significantly decreases the quality of life (QoL) for 17–29 % of the older adult population of the USA [19]. Reports of its prevalence in European countries vary, ranging from 6 % at 50 years of age and 15 % of those at 65 years of age in the Swedish population and to more than 30 % of the Hungarian population. In the English population, self-reported xerostomia was found in 63 % of hospitalized patients [20–22]. However, the prevalence of the sensation of dry mouth is as high as almost 100 % in patients with Sjögren’s syndrome and those who are receiving radiation therapy for head and neck cancers [23]. Xerostomia in patients on maintenance hemodialysis can be caused by reduced salivary flow secondary to atrophy and fibrosis of the salivary glands, use of certain medications, but mainly to the restriction of fluid intake [24].

Literature data shows that the percentage of HD patients who suffer from xerostomia is high and ranges between 32.9 and 76.4 % [4–7]. This is in accord with the present study, in which 71.8 % of the HD patients report having dry mouth symptoms. Tools such as the xerostomia inventory (XI) can not only be used to discriminate individuals with or without self-reported dry mouth, but also help to assess the severity of xerostomia. The subjective feeling of dry mouth for HD patients in the present study (XI = 33.1 ± 10.7) was found to be similar to that of HD patients according to Bots et al. (XI = 28.3 ± 9.1) [4] and higher than seen in Teratani et al. (XI = 22.2 ± 7.4 and XI = 20.6 ± 5.9 [25], in patients who need hemodialysis owing to diabetic nephropathy and chronic glomerulonephritis, respectively). The XI score was also seen to be higher than for the general population of older adults described in Wiener et al. (21.7 ± 7.4) [17].

Oral dryness is often accompanied with hyposalivation, but not always. The present study confirms those of other authors in the respect that some patients experience a subjective feeling of dry mouth despite normal, objectively measured, levels of saliva secretion, whereas others do not complain about oral dryness, despite objectively diagnosed hyposalivation [21, 26, 27]. Wiener et al. [17] report that a total of 70.4 % of the participants in their study group suffered from hyposalivation, but did not report having xerostomia. In our study, only 4 of 32 HD patients with hyposalivation did not report xerostomia, which confirms that the prevalence of xerostomia in HD patients is more frequent than in the general population of older adults with hyposalivation [17]. On the other hand, in our study, only 5 of 76 patients with a salivary flow higher than 0.1 mL/min reported never having any symptoms of dry mouth.

According to the literature, the sensation of xerostomia may occur in people who have normal salivary flow rates because areas of localized mucosal dehydration may exist in conjunction with normal salivary flow [17]. A literature search completed over the period of 1980–1999 by Mistiaen [28] describes the prevalence of thirst to vary from 6 to 95 %, but the most representative studies on relatively large samples of HD patients report it to be around 85 %. Of the groups of patients with low thirst scores, 14 % reported feeling not abnormally thirsty and 15 % never thirsty. In our study, only one patient reported a DTI score of 7 (never) for all questions concerning perceived thirst and 10.81 % with answers hardly ever and never for the rest of questions (DTI score 8 and 9 in 2 and 5 patients, respectively). The mean DTI score of the patients (18.6 ± 6.21) was comparable with that of the patients in the Bots et al. study (20.3 ± 7.3). This slightly lower value can be explained by the shorter mean time of treatment of hemodialysis in our study, which, according the Bots et al. [4] findings, may influence thirst sensation (patients >24 months on dialysis reported more thirst—DTI score 21.6 ± 7.1—than patients ≤24 months on dialysis—DTI score 18.0 ± 7.4).

The present study investigates whether hyposalivation, xerostomia or thirst sensation were related to IWG. Similar to Bots et al., a significant correlation was found between IWG and thirst, as well as IWG and xerostomia in whole group of HD patients, and no relationship between UWS flow rate and IWG was observed. Nevertheless, when the subjects were divided into groups with and without hyposalivation, the average IWG was found to be significantly higher in patients with hyposalivation, which may suggest that this factor plays an important role in enhancing weight gain. It is worth noting that in the hyposalivation subgroup, only self-reported dry mouth was related to IWG, which may indicate that mouth dryness dominates over thirst sensation in HD patients with hyposalivation, and this is the main reason for frequent fluid intake. As a very low amount of saliva causes oral mucosa dryness (dehydration), those patients frequently moisten oral mucosa by sipping fluids, which may mask the perception of thirst.

However, in the subgroup with a saliva flow rate higher than 0.1 mL/min, the thirst sensation was the one that correlated with IWG. Also, other studies confirm that thirst is related to IWG despite being based on a range of methodologies involving different answer categories varying from a dichotomous yes/no answer to 5-point answer categories or visual analogue scales (VAS) [28]. Nevertheless, Mistiaen, in a review of published studies concerning the relationship between thirst and IWG in hemodialysis patients, underlines that this relationship is not necessarily as linear as often thought. For example, patients with high IWG who do not complain of thirst may drink a lot to prevent thirst, or drink whenever they feel slightly thirsty. It may also happen that a patient feels very thirsty but are able to refrain from drinking [28].

Additionally, the concept of an individual sodium set-point and its kinetics in hemodialysis must be considered in regard to IWG and thirst or xerostomia. To maintain osmolar homeostasis, the sodium changes are always linked with water ingestion, which is of importance in the determination of the IWG [29]. The sodium water overloads must be removed during HD, but in patients with a lower sodium set-point, this process is probably slower if not disrupted [30], and ultrafiltrated sodium tends to be hypotonic, the Gibbs–Donnan effect [31], which implies that the diffusion process is responsible for final sodium tuning [32].

Overall, the problem of hyposalivation and associated xerostomia, thirst or excessive IWG seems to be more complex than previously considered. One could speculate that lower serum sodium concentration (below 140 mmol/L) and elevated sodium gradient (over 3 mmol/L) [33, 34], which are rapidly normalized during hemodialysis session due to ultrafiltration (pure water removal) and dialysis with 140 mmol/L sodium in dialysate, which increases serum sodium concentration, can initiate the process of cell dehydration. Once dehydrated, cells lose their potential to produce body fluids, including saliva. Martins et al. [35] and Bots et al. [36] confirm that the saliva of hemodialysis patients is hypertonic in comparison with the saliva of healthy people and its contact with the mucous membranes of the mouth can in fact lead to cell dehydration rather than moisturization. Our earlier study demonstrates that the decrease in sodium concentration in dialysate normalizes sodium gradient and reduces IWG [11] and should be of interest, whether or not it may have an influence on saliva secretion.

The major study limitation is its design as an observational trial, which can describe only associations but does not provide certain casual relationships.

## Conclusion

Hyposalivation is one of the factors which significantly correlates with IWG. However, its influence on thirst and mouth dryness, according to survey results, seems to be weaker than expected. Additionally, hyposalivation was found to be associated with an elevated pre-dialysis sodium gradient, which serves to clarify the connection between decreased saliva production and excessive weight gain in patients on maintenance hemodialysis, as well as its underlying cause.

Although those findings potentially introduce new aspects in the assessment of the hyposalivation etiopathogenesis, the implications of our results need to be investigated in future studies.
